# The contribution of tipping fees to the operation, maintenance, and management of fecal sludge treatment plants: The case of Ghana

**DOI:** 10.1016/j.jenvman.2021.114125

**Published:** 2022-02-01

**Authors:** Rebecca Tanoh, Josiane Nikiema, Zipporah Asiedu, Nilanthi Jayathilake, Olufunke Cofie

**Affiliations:** a International Water Management Institute-West Africa Office, PMB CT 112, Cantonments, Accra, Ghana; b International Water Management Institute-Headquarters, 127 Sunil Mawatha, Pelawatte, Battaramulla, Colombo, Sri Lanka

**Keywords:** Public private partnership, Co-treatment, Developing countries, Cost recovery, Waste stabilization ponds

## Abstract

Globally, collection of tipping fees is being promoted as a solution to sustain the operation of fecal sludge treatment plants (FSTPs). Currently, there are six large-scale FSTPs in Ghana, of which five were in operation in June 2017. In Kumasi, Sekondi-Takoradi and Tamale, fecal sludge (FS) is co-treated with landfill leachate using waste stabilization ponds (WSPs). In Tema and Accra, FS is treated using WSPs and a mechanical dewatering system coupled with an upflow anaerobic sludge blanket (UASB). The focus of this study is FSTPs and to assess how, and if, the tipping fees set by the municipalities could enable cost recovery to sustain their long-term operation. Using a questionnaire survey to interview plant managers from the public and private sectors, and directors of waste management departments, we found that the overall average operation, maintenance and management (OM&M) costs per 1000 m^3^ of treated waste (FS or FS + leachate) in 2017 were USD89 in Kumasi, USD150 in Tamale, USD179 in Tema, USD244 in Sekondi-Takoradi and USD1,743 in Accra. There were important disparities between FSTPs due to their scale, age, and level of treatment and monitoring. Currently, most FSTPs charge tipping fees that range between USD310 and USD530/1000 m^3^ of FS, averaging USD421 ± 98/1000 m^3^ of FS discharged at FSTPs. Our study also showed that the OM&M costs of large-scale intensive FSTPs cannot be sustained by relying solely on tipping fees. However, there could be potential to cover the routine expenditures associated with operating smaller FSTPs that relying on WSP technologies.

## Highlights

• FSTPs experience operational issues, partially addressable by improving management.
• Private sector contributes 92% of the costs of operating, maintaining and managing FSTPs.
• Cost recovery is achievable by small FSTPs applying nature-based technologies.
• For large FSTPs, public subsidies are needed to cover the financial gap.

## Abbreviations

AMA: Accra Metropolitan Assembly
BOD: Biochemical Oxygen Demand
COD: Chemical Oxygen Demand
EPA: Environmental Protection Agency
FSTPs: Fecal Sludge Treatment Plants
FS: Fecal Sludge
FSM: Fecal Sludge Management
GHS: New Ghana Cedi
GoG: Government of Ghana
KMA: Kumasi Metropolitan Assembly
MMDAs: Metropolitan, Municipal and District Assemblies
OSS: On-Site Sanitation Systems
OM&M: Operation, Maintenance and Management
O&M: Operation and Maintenance
PPP: Public-Private-Partnerships
PPE: Personal Protective Equipment
STMA: Sekondi-Takoradi Metropolitan Assembly
SSGL: Sewage Systems Ghana Limited
SDG6: Sustainable Development Goal 6
TaMA: Tamale Metropolitan Assembly
TMA: Tema Metropolitan Assembly
UASB: Upflow Anaerobic Sludge Blanket
USD: United States Dollar
WSPs: Waste Stabilization Ponds

## 1 Introduction

The availability of safe water and proper management of human waste are major global challenges and in response, Sustainable Development Goal 6 (SDG6) was established by the United Nations in 2015. The main aim of SDG6 is to ensure the availability and sustainable management of water and sanitation for all. But, to date, providing safe sanitation – particularly in low-income countries – and achieving SDG6 are still confronted by hurdles. Moreover 61% of the global population, especially in developing countries, still does not have access to improved safe sanitation (Mallory et al., 2020).

Globally, sewer systems and on-site sanitation systems (OSS) are the main means of managing human excreta; approximately 6.1 billion people are using improved sanitation facilities globally and 3.1 billion people out of this population rely on OSS (Rao et al., 2017). In fecal sludge management (FSM) there are various handling and treatment processes for fecal sludge (FS) in different settings. The functional elements of FSM include containment on site, emptying, transportation, treatment and final disposal or reuse (Farizal and Tammarar, 2019).

Usually, reported FS accumulation rates in septic tanks or pit latrines are from 21.9 to 106 l/cap/year and from 3 to 264 l/cap/year, respectively. The variations are difficult to predict because accumulation rates are affected by various parameters, including the capacity of the OSS tanks, the time since the last emptying, the number of users, the type of toilet interface, the groundwater table and so forth (Nikiema et al., 2017). The composition of FS is highly variable and presented in the supplemental file (SI-Section 1) for selected Ghanaian cities. Compared to conventional wastewater collected through sewers, FS displays high average values of chemical oxygen demand (COD) and total nitrogen which in Ghana was found to reach 6200 and 10,500 mg/L, respectively (Nikiema et al., 2020).

Adequate FSM is a necessity to ensure protection of public and environmental health (Harada et al., 2016). However, in low-income countries such as Ghana, FSM is often overlooked and conducted suboptimally, particularly FS collection and treatment components, due to the lack of suitable treatment infrastructure or sufficient funds to cover operation and maintenance (O&M) costs in a sustainable way, in addition to other institutional and technical capacity challenges (Murray and Drechsel, 2011; Nikiema et al., 2013; Mallory et al., 2020; Amenay et al., 2021).

There are important differences concerning the costs of sanitation services and how they are financed Dodane et al. (2012); Cairns-Smith et al., 2014; Daudey (2018). Sewer-based wastewater conveyance and treatment systems are highly subsidized, but this is usually not the case for FSM. Hence, contributions from households to cover sanitation service charges in the city of Kampala are only 14% for households connected to sewers whereas households relying on OSS typically contribute about 93% of the total capital and operating costs in FSM (McConville et al., 2019).

On average, households pay on-demand emptying and collection service providers (mostly from the private sector), amounts ranging from USD28 in Asia up to USD100 and USD233 in Africa and Latin America, respectively (Rao et al., 2016). If collection results in minimum profit because of longer travel distances and time involved coupled with the age of the collection fleet, usually little provision is made to ensure proper treatment of FS at the end of the service chain (Taweesan et al., 2015; Nikiema et al., 2020; Singh et al., 2021). This is why treatment faces most constraints in the FSM service chain (Srinivasan, 2018; Bhagwan et al., 2019).

To make FS treatment more attractive to the private sector and sustain O&M of fecal sludge treatment plants (FSTPs), a revenue stream has been created by establishing a discharge or disposal fee at treatment facilities, which is occasionally referred to as a ‘tipping fee’ (Rao et al., 2016). This measure has been promoted by the public sector along with the adoption and enforcement of the ‘polluter-pays-principle’ in the waste management sector, which many countries have adopted. The tipping fees may represent the main source of income to routinely operate and maintain the FSTP systems and are indirectly paid by households through FS emptiers. Usually, the volume of waste to be disposed determines the price of the tipping fees (Farizal and Tammarar, 2019).

Very little is known as to what extent the tipping fees can help address FS treatment costs in developing countries. This study aims at filling this gap using Ghana as a typical case. It investigates the current operation, maintenance and management (OM&M) costs of existing FSTPs in Ghana to assess how, and if, the tipping fees set by the municipalities could really enable cost recovery to sustain their long-term operation.

## 2 Materials and methods

### 2.1 The context

In Ghana, a lower middle-income economy, the management of sanitation falls under the direction of the Ministry of Sanitation and Water Resources (MSWR). Implementation is carried out by Waste Management Departments (WMDs) and/or Environmental Health Departments (EHDs) in the Metropolitan, Municipal and District Assemblies (MMDAs) (Mansour and Esseku, 2017). The Ghana Environmental Protection Agency (EPA) has set guidelines for discharged water quality, which were developed by building on World Health Organization (WHO) recommendations (SI-Section 1) (Agyemang et al., 2013; EPA, 2000). Overall, the regulatory framework is well developed but, as in many developing countries, enforcement remains weak. MMDAs act as service providers for some aspects of waste management or public toilet services, although the policy states that services should be delegated to private operators (Mansour and Esseku, 2017).

In 2010, Ghana approved the polluter-pays-principle under the government's revised environmental sanitation policy. In addition, the public sector aims at enhancing implementation of public-private partnerships (PPP) for effective delivery of environmental sanitation services. However, the success of these efforts has remained limited. To date, only 18.8% of the urban population of Ghana has access to a private and improved toilet facility; 66.4% of the urban population relies on shared facilities for sanitation, mainly through public toilets, or compound toilets. Additionally, over 92% of the toilet facilities in Ghana are connected to OSS (17.3% septic tanks and 74.8% pit latrines). Finally, 6.6% and 8.2% of the urban population in Ghana resort to unsafe sanitation means or to open defecation, respectively (JMP, 2021).

FS emptying and collection services have improved significantly in Ghana in recent times, along with the involvement of the private sector. Mechanical emptying services using vacuum trucks with a capacity of 6–18 m^3^ are commonly offered in Ghanaian cities, though affordability and travel logistics constitute a hindrance to their use. Most FS vacuum trucks are operated by individual private entrepreneurs with minimal control from the MMDAs, beyond the designation of official FS dumping sites. Manual emptying of OSS is observed in smaller-sized towns (Nikiema et al., 2020).

**Fig. 1:**
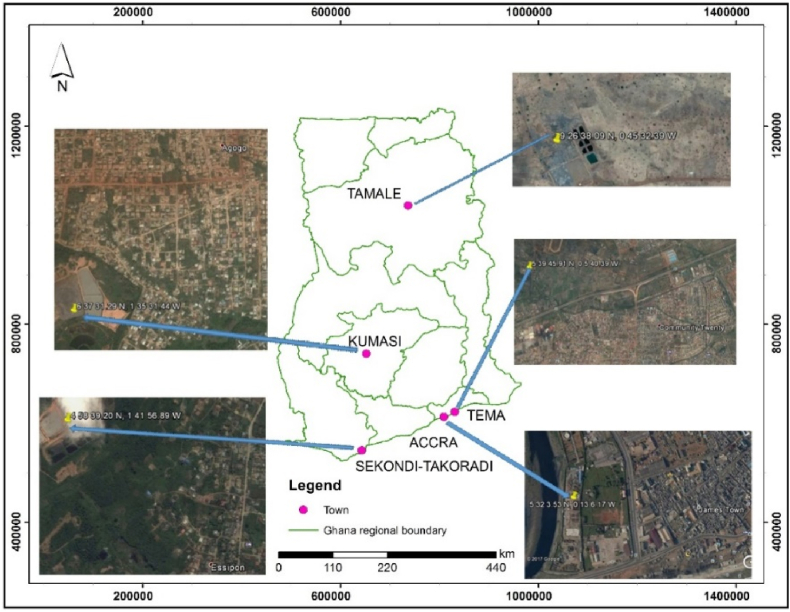
Locations of the FSTPs in Ghana included in this study as of June 2017.

Although there are improvements in FS emptying and collection, treatment is limited. Currently, six FSTPs are in operation in various locations in Ghana, but at the time of the study, there were only five (Fig. 1). In Kumasi (Ashanti Region), Sekondi-Takoradi (Western Region) and Tamale (Northern region), FS is co-treated with landfill leachate using WSPs. In Tema and Accra (both in the Greater Accra Region), FS is treated using WSPs and a mechanical dewatering system coupled with an upflow anaerobic sludge blanket (UASB), respectively. In late 2017, one FSTP using a similar and advanced UASB technology, with capacity limited to only three-quarters of that of the Accra FSTP capacity, was launched in Adjen-Kotoku (Greater Accra Region) but it was not included in this study due to insufficient data.

In order to understand the contribution of tipping fees to the OM&M of FS treatment plants in Ghana, we assessed the current performance status of FSM, estimated and compared actual costs to the expected costs of O&M, and provided recommendations for improvement.

### 2.2 Data collection and analysis

This study was carried out at all five FSTPs in operation at the time of the study, i.e. between July and November 2017 (Fig. 1). Primary and secondary data were collected. Secondary data were obtained from the literature and covered generalities on FSTPs, volumes of FS treated and treatment performance of FSTPs. Secondary data were used to cross-validate primary data. However, primary data collection took place through field observations, individual interviews and key informant discussion targeted at FSTP staff, FSTP managers with direct oversight over the FSTP plants, public and private sector actors, and directors of the waste management departments. The questionnaire used is provided in SI-Section 3. The costs of revenue were estimated (Table 1). The only source of revenue was tipping fees of which the rate was publicly known.

**Table 1:** Methodology followed in clustering of costs for this study.

To estimate	We considered	Approach
Revenues	Tipping fees	• Data were collected on the pricing model. Then the average tipping fee per 1000 m^3^ of FS was calculated. • The volume of FS deposited at the FSTP was determined and validated through field visits and published data. • Revenues were derived by multiplying the average tipping fee per 1000 m^3^ by the FS volume.
Operational costs	Equipment	• Cleaning and renewal of Personal Protective Equipment (PPE) and operation of pumps, generators, forklifts, and other equipment necessary for each FSTP.
	Others	• Utilities (water and electricity) required for FSTP operation. Also, chemicals used in processes.
Maintenance costs	Equipment	• Pumps, generators, forklifts, ponds, and any other equipment necessary for FSTP operation.
	Others	• Cost of clearing of weeds within the FSTP premises, the maintenance of other non-essential electrical appliances such as air-conditioners and any general maintenance of the premises and surroundings.
Management cost	Staff travel	• Cost of OM&M of motorcycles and cars, taxis and other professional travel costs.
	Office management	• Cost of office charges such as stationary and any other management fees.
	Staff time	• Cost of the gross salaries of all temporary and full-time FSTP staff, posted on site or otherwise.

Descriptive statistics were used to analyze the data compiled for this study. We conducted the data analysis to reveal treated volumes ranges, the specific costs of OM&M, the revenue collected through tipping fees in each FSTP, and occasionally the treatment performance when sufficient data were available (which was the case for FSTPs in Accra and Sekondi-Takoradi only) (Tables S-4 and S-5). Table 1 describes how input data were considered in the assessment. The local currency is the New Ghana Cedi (GHS), and the exchange rate was GHS1.00 = USD0.2272 in November 2017. To actualize costs reported in literature, the base year index used was: 21.12 for 2001, 100 for 2010 and 152.99 for 2014.

### 2.3 Limitations of the study

Audited accounts of the FSTPs could not be assessed hence the study was based on estimated revenue data and self-reported expenditures by the managing entities. In Tamale and Tema, some negligible costs, resulting from office operations or utilities, could not be captured as they were unknown. Exact volumes of landfill leachate co-treated with FS by the FSTP where applicable could not be assessed, hence where necessary, we used the design volume for the landfill leachate. There was no information on whether this design capacity was met, exceeded or not met in reality. Typical characteristics of leachate in Ghana are provided in SI-Section 1.

## 3 Results

The characteristics of the FSTPs studied are presented in SI-Section 1 (Tables S–4). A flowchart of the treatment process implemented at each FSTP is given in SI-Section 2. Primary data collected through our study are provided in SI-Section 4.

### 3.1 Staffing of FSTPs

**Fig. 2:**
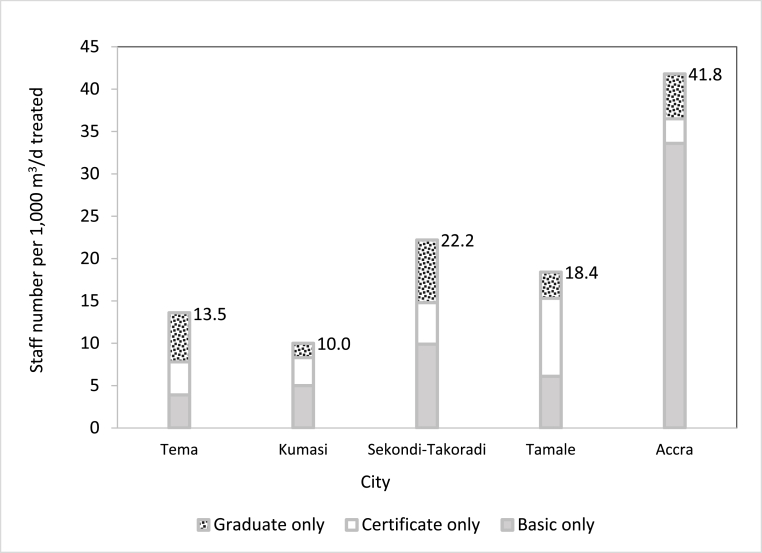
Staff ratio at FSTPs in Ghana.

As of June 2017, five FSTPs in Ghana employed 99 staff affiliated to the public or the private sectors, with 98% on a full-time basis (although in practice, only some of their time was often assigned to direct FSTP-related activities) (Fig. 2). Specifically, 68 staff members had a basic education and worked as laborers to ensure the security and cleanliness of the FSTP premises; 13 staff members had a certificate in sanitation and worked as supervisors, recorders, and reported on the number of trucks received by the plants; 18 staff members had a university degree, i.e. a bachelor's or a master's degree or a doctorate. In theory, they were given the responsibility to oversee FSTP activities, e.g. to draft quarterly reports on each FSTP OM&M.

On average, the staff ratio in Ghana was 21 ± 13 persons per 1000 m^3^/d of liquid treated (FS only or FS + leachate, based on the location) whereas there were 16 persons in the case of WSP-based FSTPs. The staff ratio (Fig. 2) in Kumasi was the lowest in Ghana (10 persons per 1000 m^3^/d of co-treated FS + leachate) while it was highest for Accra (about 42 persons per 1000 m^3^/d of FS treated).

### 3.2 Management costs of FSTPs

**Fig. 3:**
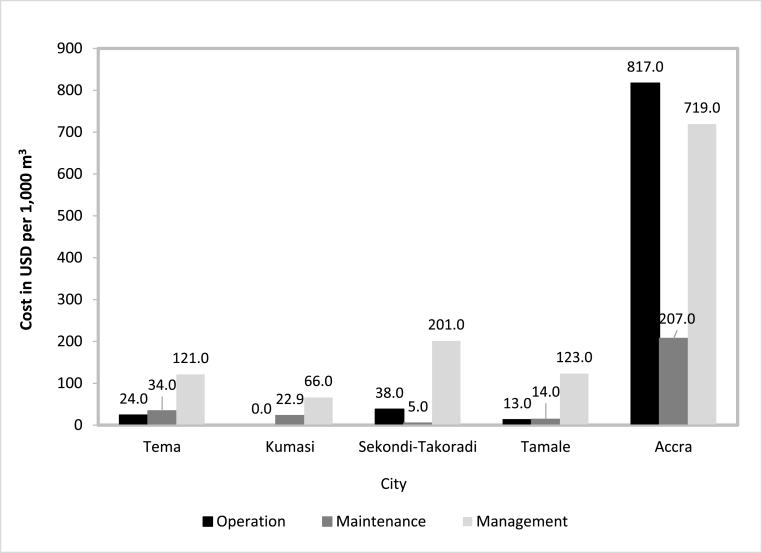
Operation, maintenance and management costs of FSTPs in Ghana.

Fig. 3 details the OM&M costs of each FSTP in Ghana. Cost values obtained for FSTP management (including staff time, staff travel as well as office management costs) ranged from USD14,495/year in Kumasi to USD446,189/year in Accra.

Among WSP-based treatment systems, the FSTP in Sekondi-Takoradi had the highest management cost of USD201 per 1000 m^3^ of co-treated FS + leachate. The lowest was in Kumasi at USD66 per 1000 m^3^ of co-treated FS + leachate. For this technology, management cost constituted staff salaries (83% of total), staff travel to the plant site for monitoring (16%), and general administration (1%). Our inspections confirmed that many on-site offices were not in use as planned (they lacked basic infrastructure to function adequately including electricity sometimes). Motorcycles were generally used by the technicians for movement on site or off site, but cars were used by higher grade staff (See Tables S–7).

The FSTP in Accra deviated from this trend. In Accra, the management cost was USD719 per 1000 m^3^ of FS treated. About 85% of the total management charge of Ghana was incurred in Accra, for a treated volume of FS limited to 48% of the total volume of liquid (i.e. FS + landfill leachate) (co)treated by FSTPs in-country. The high staff ratio recorded in Accra translated into staff salaries reaching 51% of the total management cost. Also, in Accra, the FSTP offered a shuttle service for staff to commute to work. Travel costs represented 6% of the total management cost. Finally, general administration costs represented 43% of the total management cost incurred by the FSTP.

On a national average, the management cost within the FSTPs in Ghana was USD246 ± 269 per 1000 m^3^ of FS (co)treated.

### 3.3 Operation costs of FSTPs

Operation costs included costs of utilities, chemicals used in the processes, PPE cleaning and renewal, and other costs of equipment necessary for operating each FSTP to function as designed. The institutional arrangements to cover the operation costs varied from one location to the other. The operation costs were routinely covered by the public sector in Tema and Tamale, co-shared by public and private parties in Kumasi and Sekondi-Takoradi or paid in full by the private party (namely SSGL) in Accra. In the latter scenario, our interviewees revealed that SSGL was supposed to receive quarterly payments (amounts not disclosed) for the service they provided, to cover the OM&M of the FSTP they managed. In Kumasi, an agreement was signed between the municipality (KMA) and one private company for the management of the FSTP. However the operation of the plant was constrained due to cash flow problems. In Sekondi-Takoradi, the private contractor who also managed the landfill paid for the renewal of the PPEs, the analysis of effluent quality, and the cleaning of equipment while the STMA paid the utility bills (electricity and water). For this scenario, additional details on the PPP arrangement were not available to the research team. In Tema and Tamale, there were no private parties involved in the operation of the FSTPs.

For the four WSP-based FSTPs, the flow of the liquids from one pond to another was solely by gravity and pumps were not required constantly. This was however very different for the FSTP in Accra, which was highly mechanized and consumed significant amounts of electric power and chemicals during the dewatering step.

FSTPs in Ghana spent between USD1,499 (in Tamale) and USD507,110 (in Accra) each year on operation. Consequently, the cost of operations for WSP-based FSTPs ranged from USD13 per 1000 m^3^ (in Tamale) to USD38 per 1000 m^3^ (in Sekondi-Takoradi) (Fig. 3). In Tema, the operation cost was USD24 per 1000 m^3^ of FS while no operation cost was reported for Kumasi due to limited O&M activities with no recorded data. However, in Accra where the UASB technology was used, the operation cost was USD817 per 1000 m^3^ of FS, i.e. over 20 times that of Sekondi-Takoradi costs. The total operation costs of the WSP-based FSTPs recorded for all the four locations was USD11,792/year, i.e. 2.3% of the overall total cost of operation of FSTPs in Ghana. The average cost of operation of the FSTPs in Ghana was USD223 ± 396 per 1000 m^3^.

WSP-based FSTPs faced challenges with quality of treatment although the effluent of the FSTP in Sekondi-Takoradi met many of the key effluent quality discharge criteria set by EPA Ghana, including biochemical oxygen demand (BOD) which was only 35 mg/l (SI-Section 1).

### 3.4 Maintenance costs of FSTPs

In all the FSTPs, the main maintenance activities involved the desludging of the ponds, the clearing of the access road, weed removal from the premises, and the servicing of equipment used for the FS treatment process. Each FSTP in Ghana spent from USD654 (in Sekondi-Takoradi) to USD128,140 (in Accra) each year on maintenance activities.

Overall, the average cost of maintenance of FSTPs in Ghana was USD56 ± 85 per 1000 m^3^ of liquid waste (either FS only or FS + leachate). For the WSPs, the cost of maintenance ranged from USD5.00 (in Sekondi-Takoradi) to USD34.00 (in Tema) per 1000 m^3^ of FS or FS + landfill leachate (Fig. 3). In Sekondi-Takoradi, the FSTP relied mainly on existing full-time staff and machinery for weed removal and cleaning activities while in Tema, Kumasi, and other locations, costs for equivalent activities were consistently higher, as these facilities resorted to casual and more expensive labor. This maintenance cost was also affected by the size of the premises to be maintained. Some savings through cost sharing could also be achieved when the landfill and FSTP were located at the same place.

In Accra, the maintenance cost was USD207 per 1000 m^3^, i.e. over six times more than that of Tema (the highest among WSP-based FSTPs). The combined maintenance cost for the four WSPs was only USD13,611/year, i.e. 10% of the total cost of maintenance of FSTPs in Ghana (the rest was from the Accra FSTP). However, these plants had a combined capacity similar to that of Accra, which demanded much more and was compact. For WSPs, desludging of ponds represented, on average, 18% of the total maintenance cost while other maintenance activities such as removal of weeds, rehabilitation of the access road, cleaning of drains, and renewal of cleaning tools corresponded to 82% of the total maintenance cost. For the Accra FSTP, servicing of pumps, the generator, and other plant equipment corresponded to 96% of the maintenance cost. The remaining 4% was for servicing non-technical equipment such as air-conditioners.

### 3.5 Tipping fees

**Fig. 4:**
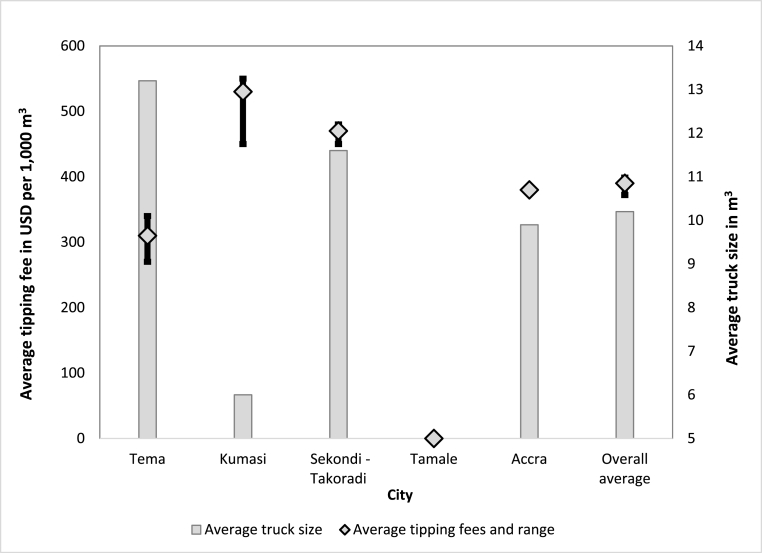
Average tipping fees and truck size at FSTPs in Ghana.

Fig. 4 details the tipping fees generated by the FSTPs. In principle, these fees were collected to cover at least in part the charges incurred for the safe processing of the FS. In practice, the tipping fees were often seen as a general revenue stream for the MMDAs and were therefore not 100% dedicated to the O&M of FSTPs. In the FSTPs co-treating nearby landfill leachate with the FS, tipping fees were not charged for the landfill leachate treatment. However, when a private entity was engaged in the management of the solid waste, it could have responsibility for covering some of the expenditures that also benefited FS treatment. However, it was unclear if the value of these services was equitable compensation compared to the cost of leachate management.

Tipping fees were collected in most Ghanaian municipalities, with the exception of Tamale. In Tamale, the waiving of tipping fees was ‘temporarily’ implemented to incentivize truck drivers to discharge at the FSTP site. Most MMDAs adopted a tariff structure that depended on the theoretical capacity of trucks. Though higher charges were collected for larger trucks, the ratio of tipping fee to truck volume was usually reduced with increase in truck size. In all cases, collection of tipping fees was the responsibility of the public sector, even when a private company was also involved in or in charge of the OM&M of FSTPs. When applicable, private companies were expected to be paid exclusively by the public entities, and the amounts involved were based on their agreements. But the case of Kumasi and to some extent Accra showed that this was not done without challenges.

The tipping fees collected in Ghana ranged between USD310 and USD530 per 1000 m^3^ of FS. The average tipping fees applied in Ghana corresponded to USD421 ± 98 per 1000 m^3^ of FS. Annual revenues made on average by FSTPs were USD234,963 in Accra (excluding electricity generation from biogas which was only achieved for internal use) and ranged from 0 to USD58,306 for each of the other cities. In total, the revenue generated from tipping fees for FS treatment in Ghana should amount to USD386,387 yearly.

Detailed cost data per plant are provided in SI-Section 5.

## 4 Discussion

In Ghana, with only five engineered FSTPs as of June 2017, about 2870 m^3^ of FS are processed daily, along with an estimated 680 m^3^ of landfill leachate, making a total of approximately 3550 m^3^ of non-sewered liquid waste treated daily. Compared to older plants which are overloaded, FSTPs that were constructed recently (i.e. Accra and Sekondi-Takoradi which were 1 and 5 years old, respectively, at the time of the study) process FS volumes that align with their design capacity, and thus possess increased chances of good treatment performance. However, FSTPs that have been in operation for a longer period experience technical issues, from disrepair of some equipment, which was never replaced when it broke down (e.g. weighing bridges), to malfunctioning of some process components (e.g. screens, ponds). Most plants experience issues with odor, resulting in complaints by neighboring communities (SI-Section 6). The average OM&M cost in Ghana, considering the five FSTPs included in this study, was USD525 ± 777 per 1000 m^3^ of treated FS only or FS + leachate, based on location.

Table 2 presents a summary of the institutional arrangements and business models for each FSTP.

**Table 2:** Management structures and business models of Ghana FSTPs, as of 2017.

City (municipality)	Construction funds origin	Current business model	Role of the public sector	Role of the private sector	Ratio: revenues/OM&M costs
Kumasi (KMA)	Public funds	• FSTP staff salaries paid by Government of Ghana (GoG) • Other OM&M fees paid from tipping fees	• Full control over the plant OM&M • Collection of tipping fees	The PPP signed earlier was not enforced	2.98
Accra (AMA)	Private funds	• Service fee paid by AMA	• Collection of tipping fees • Payment of a service fee to the private entity	• Full control over FSTP OM&M • A PPP SSGL-AMA was in place	0.22
Tema (TMA)	Public funds	• FSTP staff salaries paid by GoG • Other OM&M fees paid from tipping fees	• Full control over the plant • Collection of tipping fees	Not applicable	1.71
Sekondi-Takoradi (STMA)	Public funds	• FSTP staff salaries paid by GoG • PPEs and sample analysis paid by the private entity • Other OM&M fees paid from tipping fees	• It covered 36% of the operation costs, all management and maintenance costs, or 90% of the OM&M costs • Collection of tipping fees	‘Waste Landfills’ managed the nearby landfill and covered 10% of the FSTP OM&M costs	0.97
Tamale (TaMA)	Public funds	• FSTP staff salaries paid by GoG • Other OM&M fees paid from tipping fees	• Full control over the plant • Collection of tipping fees waived	Not applicable	0.00

### 4.1 Comparing OM&M costs in Ghana to other experience

#### 4.1.1 WSP-based treatment

Nature-based treatment systems for FS treatment are common technologies in many countries. But it is usually difficult to compare treatment costs for FS given the high variability in its characteristics (Bassan and Robbins, 2014). Other factors influencing differences in OM&M costs can be linked to local contexts (electricity rates and staff costs), scale of the treatment plants, etc. (Singh et al., 2017).

Our study revealed that the WSP OM&M costs per 1000 m^3^ were USD89 in Kumasi, USD150 in Tamale, USD179 in Tema, and USD244 in Sekondi-Takoradi, compared to a global average of USD171 ± 48. There were significantly below values published from other studies, probably because of their insufficient performance. In 2001, the OM&M cost of WSPs used in FS treatment globally was USD410 ± 180 (or about GHS300 ± 130) per 1000 m^3^ of FS treated in FSTPs having 50–170 m^3^/d of treatment capacity (Steiner et al., 2002). Actualization to 2014 gives a corresponding value of USD490 ± 220/1000 m^3^ (GHS2,060 ± 550). Considering plants in Ghana only (with capacities of between 80 and 170 m^3^ FS/d), OM&M cost in 2001 averaged USD320 ± 180 per 1000 m^3^ of FS treated (GHS230 ± 130) (Steiner et al., 2002), actualized to USD390 ± 210 (GHS1,640 ± 710) as of 2014. These OM&M costs are in the same range as the tipping fees collected in Ghana in 2017.

Costs of FSTPs in Ghana are also much lower than in other countries, e.g. India, where they ranged from USD1,140 to USD3,510 per 1000 m^3^ for FSTPs with capacity of 50 and 12 m^3^/day, respectively (Saket, 2017). Various factors can explain the cost difference with Ghana. Beyond economies of scale, the FS content and the treated water quality standards are different in India compared to Ghana (e.g. standards for BOD are 20 mg/l instead of 50 mg/l in Ghana) (EPA, 2000; CPCB, 2013; Sagoe et al., 2019). Polishing treatment units, in addition to the conventional nature-based treatment system, are therefore commonly considered in India.

#### 4.1.2 Mechanical dewatering plus UASB

The prevalence of mechanical treatment technology in Accra over nature-based systems such as WSPs is commonly observed in large urban areas, where volumes of wastes are high and space availability is limited (Navrekar, 2016). But this results in higher OM&M costs; in Ghana, they were USD1,743 per 1000 m^3^ of FS, which was 4.4 times higher than that of the most expensive WSP. In Ghana, there is the opportunity to produce electricity, although in practice this was not achieved for months after commissioning the plant in Accra.

The treatment system established in Accra was not conventionally applied to FS treatment. Therefore, it was not possible to find comparative plants. In India, a UASB treating 1000 m^3^/d of sewered wastewater costs 3.1 times more than the equivalent WSP (Khalil et al., 2008). Logically, this ratio increases when the capacity of the WSP is lowered. In Ghana, our study found that the UASB process cost, respectively, at least 3 and 7 times more than under optimal and under the current maximum OM&M for the WSP, which was in line with the expected trend.

### 4.2 Cost recovery and role of the private sector in Ghana

In Ghana, we estimated through this study that funds spent for OM&M of FSTPs totaled USD1,188,659 each year. Only about 9% of these funds came directly from the public sector while the balance was sourced from private companies, which may in turn be paid via subsidies through different contractual arrangements that often remain undisclosed (Table 2). This high private sector contribution was mostly driven by the FSTP in Accra which was the largest (treated 60% of the collected FS in the country or 48% of the non-sewered liquids treated by the studied FSTPs), and the most expensive to run and was 100% pre-financed by a private entity. However, when focusing solely on the remaining WSPs, the actual amount spent on OM&M dropped to USD107,219. The private sector contributed only 3% of this and involvement was barely sustained. The estimated collected tipping fees were USD151,423 and exceeded the actual total expenditures by 41%, while the treatment performance of these FSTPs remained unsatisfactory.

In most developing countries, private sector involvement is mostly limited to the role played by small-scale independent providers who fill financially profitable niches, e.g. FS emptying, and operating pay-to-use toilet blocks (Murray et al., 2011; Taweesan et al., 2015). Governments are increasingly looking to engage the private sector in FS treatment although the interest of the latter is constrained because its perceived business prospects are not attractive. Therefore, the situation in Ghana is rather unusual and is likely driven by the service agreements signed by the government with these parties under undisclosed terms, which is not uncommon in the waste management sector, globally.

Under the scenarios described before, the FSTPs of Tamale and Accra are not able to achieve notable cost recovery to sustain their OM&M. In Tamale, no revenue is generated due to the waiving of the tipping fees while in Accra, the OM&M of the plant costs 4.6 times more than the collected tipping fees, meaning an OM&M cost recovery of 22%. However the plants in Tema and Kumasi are profitable with revenues being on average 71% and up to 198% higher than costs currently incurred, respectively, but the two FSTPs do not perform satisfactorily. The FSTP of Sekondi-Takoradi is the best example in Ghana, with a nearly balanced financial budget (97% of OM&M cost recovery) while the performance of the FSTP for now meets many key water-quality criteria, including BOD.

In Ghana, policy aims at FSTPs becoming financially independent in terms of their operational costs and for services to be delivered by the private sector. In the current scenarios, the private sector can only be engaged for the OM&M of already constructed FSTPs, provided the private sector actors are allowed to collect tipping fees directly from truck operators. But this would not enable recovery of depreciation costs for the FSTP, which would still require public funds/subsidies. This financial model would work for some nature-based plants but not for intensive plants, which may be necessary for large cities. Financial models such as build-operate-transfer (BOT), build-own-operate (BOO) or partial privatization are unrealistic for implementation without significant subsidization.

Involvement of the private sector for plant OM&M could bring several benefits as plant management would be assigned to entities with adequate technical capacity. This could help to address some of the gaps of the current models which entrust FSTP OM&M to municipalities, e.g. by optimizing plant staff structure, to better align it with the FSTP needs, leading to cost savings. In addition, monitoring of activities and treatment performance indicators would have to be achieved by the public sector.

### 4.3 Possible solutions to bridging FSTP financial gaps for expensive plants

Policies in Ghana want to attract the private sector into FSTP OM&M through PPP. This paper shows that tipping fees are an important addition to FSTP management. Revenues from tipping fees could be utilized to co-fund the OM&M of FSTPs, create opportunities for local employment, sustain FSTP operations, and reduce risks for private entities, if these revenues are available. Tipping fees could also help to co-fund other common post-treatment activities (such as valorization of FSTP by-products), although recycling of FSTP by-products would result in higher OM&M cost for both the FSTP and the marketing of FS-based recycled products, which would also have to be offset by sales of high-quality products (Nikiema et al., 2020; Mansour et al., 2021).

More precisely, there is a great opportunity to address the dual challenge of waste management and soil nutrient depletion in developing countries via the safe recovery of nutrients and organic matter from both solid and liquid waste streams for reuse in agriculture (Gwara et al., 2020). Commercialization of waste-based organic fertilizers such as Fortifer™ (i.e. a mineral-enriched co-compost of FS and organic solid waste) has the potential to generate significant benefits for developing economies via cost recovery for the sanitation sector and providing affordable alternative agricultural inputs for smallholder farmers. Nevertheless, marketing of such composts is often not profitable and sustainable as it requires specific expertise which may be missing in municipalities and technical waste processing firms (Mallory et al., 2020). Moreover, marketing of such compost may be integrated in other innovative solutions which requires adaptive and flexible approach beyond the confines of waste management. Otoo et al. (2018) published a report to provide insights into the market demand and diffusion of the Fortifer™ product to inform businesses on the types of marketing and pricing strategies to implement in order to facilitate market entry and mitigate the effects of competition. The analyses they reported were conducted in the Greater Accra Region and Western Region in Ghana; Kampala, Uganda; Bangalore, India; Hanoi, Vietnam; and Kurunegala, Sri Lanka. Factors such as availability of credit-based transactions, price of the product, nutrient content, and application methods were all product attributes, which could help in promoting adoption across all the study areas. Analysis of farmers’ perceptions about Fortifer™ in all the study areas, suggested that adoption would only occur if it is certified by trusted third-party entities (preferably a governmental authority), which is the case in Ghana. OM&M break-even time for such FSTPs integrating recycling was estimated to be between three and 5 years at least (Mansour et al., 2021).

Other business models propose development of FSTP by-products into construction or several energy-based solutions, such as fuel for domestic (e.g. charcoal briquettes) or industrial use, which typically helps to recover 17.3 MJ/kg from dewatered FS, comparable with energy from other biomass fuels (Semiyaga et al., 2015; Gold et al., 2015; Mukherjee and Chakraborty, 2016; Kehrein et al., 2020; Koley, 2021). Another opportunity, when applicable, is biogas production following anaerobic decomposition which helps to enhance the energy potential of FS and results in high-value energy resources such as electricity (Semiyaga et al., 2015; Harada et al., 2016; Awuah et al., 2020; Amenay et al., 2021). The UASB process is interesting in this context if energy generation during FS treatment is productive. It is to be noted that successful and well-demonstrated business models for FSTPs are scarce. A decision on solutions to be considered should be done on a case-by-case basis, considering local opportunities and synergies to reduce costs (Otoo and Drechsel, 2018). Mechanisms to mitigate risks associated with recycling waste-based products also need to be in place (Hiremath et al., 2020).

## Credit author statement

The authors confirm contribution to the paper as follows. **Rebecca Tanoh**: Data collection, Analysis and interpretation of results, Writing- Original draft preparation. **Josiane Nikiema**: Conceptualization, Methodology, Supervision, Writing-analysis and interpretation of results, Original draft preparation, Writing- Reviewing and Editing. **Zipporah Asiedu**: Writing- Reviewing and Editing. **Nilanthi Jayathilake:** Analysis and interpretation of results, Writing- Original draft preparation. **Olufunke Cofie:** Writing- Reviewing and Editing. All authors reviewed the results and approved the final and revised version of the manuscript.

## Declaration of competing interest

The authors declare that they have no known competing financial interests or personal relationships that could have appeared to influence the work reported in this paper.
