# Supplementary material for: The contribution of tipping fees to the operation, maintenance, and management of fecal sludge treatment plants: The case of Ghana
Source: J Environ Manage. 2022 Feb 1;303:114125. doi: 10.1016/j.jenvman.2021.114125 (PMC8759011; doi:10.1016/j.jenvman.2021.114125)
Supplement: Multimedia component 1 [file mmc1.docx]

Supporting information

title:

THE CONTRIBUTION OF TIPPING FEES TO THE OPERATION, MAINTENANCE, AND MANAGEMENT OF FECAL SLUDGE TREATMENT PLANTS; THE CASE OF GHANA

Content

[1 Tables – General data 3](#_Toc86721326)

[2 Flowcharts of FSTP in Ghana 10](#_Toc86721327)

[3 Key informant questionnaire 15](#_Toc86721328)

[4 Primary Data collected 20](#_Toc86721329)

[5 Summary of OM&M cost/distribution and revenues of FSTPs in Ghana 26](#_Toc86721330)

[6 Outcome of the Self-assessment of challenges incurred by the municipalities managing FSTPs 29](#_Toc86721331)

[7 References 31](#_Toc86721332)

List of Tables

[Table S-1.Characteristics of different types of FS dumped in Ghana. 3](#_Toc86721412)

[Table S-2. Wastewater and EPA Ghana/WHO guidelines (2000/2011) 4](#_Toc86721413)

[Table S-3.Typical characteristics of landfill leachate in Greater Accra. 5](#_Toc86721414)

[Table S-4.Characteristics of FSTPs in Ghana, as of June 2017 6](#_Toc86721415)

[Table S-5. Effluent water quality for the FSTP in Sekondi – Takoradi. 8](#_Toc86721416)

[Table S-6. Effluent water quality for the FSTP in Accra. 9](#_Toc86721417)

[Table S-7.Tasks assigned to FSTP staff and their salaries 20](#_Toc86721418)

[Table S-8. Travel and office costs related to management of FSTPs (in USD) 22](#_Toc86721419)

[Table S-9. Detailed operation cost in USD/year of FSTPs in Ghana 23](#_Toc86721420)

[Table S-10. Maintenance cost of FSTPs in Ghana 25](#_Toc86721421)

[Table S-11. Summary of OM&M cost/distribution and revenues of FSTPs in Ghana 26](#_Toc86721422)

[Table S-12. Self-assessment of challenges incurred by the municipalities in managing FSTPs. 29](#_Toc86721423)

list of figures

[Figure S-1. Flowchart of the treatment process implemented at the FSTP in Tema. 10](#_Toc86721337)

[Figure S-2. Flowchart of the treatment process implemented at the FSTP in Kumasi. 11](#_Toc86721338)

[Figure S-3. Flowchart of the treatment process implemented at the FSTP in Sekondi-Takoradi. 12](#_Toc86721339)

[Figure S-4. Flowchart of the treatment process implemented at the FSTP in Tamale 13](#_Toc86721340)

[Figure S-5. Flowchart of the treatment process implemented at the FSTP in Accra 14](#_Toc86721341)

# Tables – General data

Table S-1.Characteristics of different types of FS dumped in Ghana.

| City in Ghana | Origin |  | pH | Conductivity | TS | COD | BOD | NO_3_-N | NH_3_-N | TN | TP | TK | *E. coli* | TC |
| --- | --- | --- | --- | --- | --- | --- | --- | --- | --- | --- | --- | --- | --- | --- |
|  |  |  | pH unit | S cm^-1^ | mg l^-1^ | mg l^-1^ | mg l^-1^ | mg l^-1^ | mg l^-1^ | mg l^-1^ | mg l^-1^ | mg l^-1^ | *E+05 CFU 100 ml^-1^* | *E+05 CFU 100 ml^-1^* |
| Accra^3^ | Household | Av. | 7.5 | 6,326 | 7,103 | 9,378 | 1,969 | 2.1 | 529 | 1,320 | 176 | 196 | 52.8 | 1,517 |
|  |  | SD | 0.2 | 3,127 | 5,153 | 8,212 | 1,493 | 1.5 | 233 | 239 | 124 | 137 | 25.6 | 1,202 |
|  | Public | Av. | 7 | 9,628 | 29,727 | 20,953 | 3,331 | 2.8 | 797 | 3,008 | 856 | 258 | 331 | 1,517 |
|  |  | SD | 0.3 | 1,269 | 26,090 | 13,561 | 2,326 | 3.4 | 191 | 1,932 | 679 | 121 | 277 | 1,202 |
| Tema^4^ | Household | Av. | 7.5 | 3,331 | 4,789 | 6,935 | 1,278 | 2.5 | 1,572 | 1,720 | 146 | 92 | 10.0 | 273 |
|  |  | SD | 0.1 | 1,748 | 1,275 | 2,618 | 408.5 | 2.5 | 1,752 | 1,635 | 67 | 34 | 2.5 | 335 |
|  | Public | Av. | 7.7 | 9,388 | 34,230 | 28,248 | 4,164 | 5.2 | 1,304 | 10,410 | 414 | 620 | 34.0 | 170 |
|  |  | SD | 0.3 | 8,728 | 12,232 | 12,395 | 1,083 | 5.6 | 293.8 | 11,396 | 298 | 342 | 20.4 | 154 |
| Sekondi-Takoradi^4^ | Household | Av. | 8.0 | 2,900 | 3,245 | 4,650 | 1,080 | 15.2 | 472 |  | 2.0 | 7.5 |  |  |
|  |  | SD | 0.2 |  |  |  |  |  |  |  |  |  |  |  |
|  | Public | Av. | 7.9 | 27,350 | 37,200 | 26,600 | 6,180 | 17.7 | 577 |  | 0.9 | 5.9 |  |  |
|  |  | SD | 0.4 |  |  |  |  |  |  |  |  |  |  |  |
| Yilo-Krobo ^5^ | Household | Av. | 7.8 | 2,570 | 1,170 | 1,620 | 338 | <0.001 | 420.0 | 265 | 124.5 | 28.5 | 6.0 | 16.0 |
|  |  | SD | 0.0 | 57 | 156 | 1,239 | 290 |  | 29.7 | 226 | 2.1 | 12.8 | 7.0 | 14.0 |
|  | Public septic tank | Av. | 7.4 | 7,220 | 8,440 | 5,984 | 1,315 | <0.001 | 526.5 | 1,284 | 134.0 | 165.5 | 10.0 | 16.0 |
|  |  | SD | 0.1 | 163 | 2,942 | 973 | 154 |  | 45.6 | 348 | 1.4 | 0.4 | 14.0 | 14.0 |
|  | Public pit latrine | Av. | 8.0 | 34,500 | 29,100 | 20,080 | 3,862 | <0.001 | 471.0 | 2,832 | 135.0 | 515.0 | 10.0 | 30.0 |
|  |  | SD |  |  |  |  |  |  |  |  |  |  | 14.0 | 10.0 |

Table S-2. Wastewater and EPA Ghana/WHO guidelines (2000/2011)

| Parameters | Units | Treated wastewater | |
| --- | --- | --- | --- |
|  |  | EPA standards (2000) | WHO guidelines (2011) |
| pH |  | 6-9 | 6-9 |
| TDS | mg/l | < 1,000 |  |
| Conductivity | µS/cm | 750 | 50-1,500 |
| TSS | mg/l | < 50 | 25 |
| Turbidity | mg/l | 75 | 250 (NTU) |
| BOD | mg/l | < 50 | 50 |
| COD | mg/l | < 250 | 150 |
| Cadmium | mg/l | < 0.02 |  |
| Copper | mg/l | 1 | 1 |
| Lead | mg/l | <1 |  |
| Total coliforms | MPN per 100 ml | 400 | 1,000 |
| *E. coli* | CFU per 100 ml |  | 1.00E+03 |

Table S-3.Typical characteristics of landfill leachate in Greater Accra.

| City in Ghana (name of landfill) |  | pH | Conductivity | COD | BOD | NO_3_- | TC | TDS | PO_4_- | Cd | As | Cu | Fe | Zn | Al |
| --- | --- | --- | --- | --- | --- | --- | --- | --- | --- | --- | --- | --- | --- | --- | --- |
|  |  | pH unit | μS cm^-1^ | mg l^-1^ | mg l^-1^ | mg l^-1^ | *E+05 CFU 100 ml^-1^* | mg l^-1^ | mg l^-1^ | mg l^-1^ | mg l^-1^ | mg l^-1^ | mg l^-1^ | mg l^-1^ | mg l^-1^ |
| Accra (Oblogo)^6^ | Av. | 8.5 | 5,400 | 132 | 1,881 | 5.9 |  |  | 1.0 | 0.2 | 0.2 | 11.4 | 4.9 | 6.0 | 11.8 |
|  | SD | 0.1 | 3,917 | 33 | 994 | 0.4 |  |  | 0.3 | 0.0 | 0.1 | 3.4 | 1.5 | 2.0 | 1.3 |
| Accra (Oblogo)^7^ | Av. | 7.4 | 21,100 | 1.4 | 0.5 | 12.0 |  | 11,600 | 17.5 | 1.0 |  |  | 9.3 | 0.09 |  |
|  | SD | 0.4 | 6,500 | 0.4 | 0.4 | 15.2 |  | 1,000 | 3.6 | 0.8 |  |  | 4.6 | 0.10 |  |
| Accra (four different landfills)^8^ | Min. |  |  |  |  |  | 0.64 |  |  | 0.0013 |  | 0.006 |  |  |  |
|  | Max. |  |  |  |  |  | 47.4 |  |  | 0.0126 |  | 1.018 |  |  |  |
| Accra (Sarbah) ^9^ |  | 8.97 | 3,330 | 6,672 | 220 | 5.0 | 260.0 | 16,725 | 16.3 | <0.002 |  | 0.09 | 5.0 | 0.76 |  |

Table S-4.Characteristics of FSTPs in Ghana, as of June 2017

| **Cities** | **Treatment process and description** | **Number of ponds (An-F-Ae)^1^** | **FS (landfill leachate) design capacity** | **Discharged FS range (average)** |
| --- | --- | --- | --- | --- |
| Tema | - WSP process (see Figure S-1). The treatment system included four drying beds which had never been used. - OM&M done by Tema Metropolitan Assembly (TMA). - Operated since 2004. Partial rehabilitation in 2011-2012 (World Bank funds). - Treated FS types: 70% households, 20% institutional (e.g. public toilets), and 10% industrial; 54% of the desludging trucks had capacity of 10 m^3^ while 46% had capacity of 17 m^3^. - No effluent quality monitoring done. - The TMA collected tipping fees to operate the FSTP. | 8 (4-1-3) | 250 (0) m^3^/day | 260-780 (520) m^3^/day) |
| Kumasi | - WSP process (see Figure S-2). - Initially, OM&M done jointly by Kumasi Metropolitan Assembly (KMA) and ‘J. Stanley-Owusu Group of Companies’ (JSO). Now, only managed by KMA. - Operated since 2004. Rehabilitation needed on parts of the plant; some planned to be funded by a Water and Sanitation for the Urban Poor (WSUP) project. - 30% of the desludging trucks at this site had 5 m^3^ capacity, 60% had 6 m^3^ capacity, while 10% had 8 m^3^ capacity. - No effluent quality monitoring done. - The KMA collected tipping fees to operate the FSTP. | 9 (6-1-2) | 300 (300) m^3^/day | (300) m^3^/day |
| Sekondi-Takoradi | - WSP process (see Figure S-3). - OM&M co-handled by the Sekondi-Takoradi Metropolitan Assembly (STMA) and a private company ‘Waste Landfills’ that managed the nearby landfill. - Operated since 2012. - Treated FS types: 40% households, 60% other (e.g. public toilets); 30% of the desludging trucks had 10 m^3^ capacity, 60% had 12 m^3^ capacity, while 10% had 14 m^3^ capacity. - Annual TP performance monitored yearly; to date, fairly good for key quality parameters. - The STMA collected tipping fees to operate the FSTP. | 9 (6-1-2) | 300 (200) m^3^/day | 184-225 (204) m^3^/day) |
| Tamale | - WSP process (see Figure S-4). - OM&M handled by the Tamale Metropolitan Assembly (TaMA), serving both the TaMA and Sagnarigu District. - Operated since 2005. Rehabilitated (access road and drainage) in 2016. - Treated FS origin: 70% households, 30% institutional (e.g. public toilets). Information on truck sizes not recorded. - No effluent quality monitoring done. No treated effluent discharge done. - The TaMA did not collect tipping fees to operate the FSTP. | 7 (2+1 control pond -4-1) | 420 (180) m^3^/day | 93-280  (147) m^3^/day |
| Accra | - Mechanical FS dewatering + 4 UASB reactors (see Figure S-5). - Constructed and managed by the Sewerage Systems Ghana Ltd. (SSGL) Company under a PPP with Accra Metropolitan Assembly (AMA). - Operated since December 2016. - FS origin (Greater Accra): 42% households, 57% institutional and 1% industrial; 70% of the trucks had about 9 m^3^ capacity while 30% had >12 m^3^ capacity. - Regular TP performance monitoring on site. - Water reuse planned, but not yet achieved: 10% for irrigation of lawns, 60% for FSTP operations (washing, drainage), and 30% discharged into the environment. - The AMA collected tipping fees. Later it paid a fee to SSGL (amount undisclosed). | Not applicable | 2,000 (0) m^3^/day | 1,050-2,300 (1,700) m^3^/day |

^1^ Anaerobic-facultative-aerobic.

Table S-5. Effluent water quality for the FSTP in Sekondi – Takoradi.

| **Target parameter** | **Effluent average concentration^10^** | **Removal rate (%)^10^** | **EPA (2000)/WHO (2011)**  **Guidelines^1,2^** | **Treatment performance** |
| --- | --- | --- | --- | --- |
| Total solids (mg/l) | 155.4 | 95.05 | 25 | Insufficient^a^ |
| BOD (mg/l) | 35 | 92.9 | <50 | Satisfactory |
| pH | 8.06 |  | 6-9 | Satisfactory |
| *E. coli* (MPN/100 ml) | 1,284 |  | 1,000 | Slightly insufficient |
| Turbidity (NTU) | 145 |  | 75 | Insufficient |
| Metals: Cu (mg/l) | 0.051 |  | 1 | Satisfactory |
| Metals: Zn (mg/l) | 0.0324 |  | 3 | Satisfactory |

^a^ The treatment performance is insufficient because it does not meet the Ghana EPA guidelines, despite its good TS removal rate (95%).

Table S-6. Effluent water quality for the FSTP in Accra.

| **Target parameter** | **Units** | **Average concentration in effluent^11^** | **Removal rate (%)^11^** | **EPA (2000)/WHO (2011) guidelines ^1,2^** | **Treatment performance** |
| --- | --- | --- | --- | --- | --- |
|  |  |  |  |  |  |
| Total solids | mg/l | 30 | 70-94 | 50 | Satisfactory |
| COD | mg/l | 180 | 60-92 | 250 |  |
| BOD | mg/l |  |  |  |  |
| Ammonia | mg/l | 1.2 |  | ≤1 | Insufficient |
| Total nitrogen | mg/l | 1.0 |  | ≤1 | Satisfactory |
| Total phosphorus | mg/l | 1.3 |  | ≤5 | Satisfactory |

# Flowcharts of FSTP in Ghana

MATURATION POND

MATURATION POND

ANAEROBIC POND

ANAEROBIC POND

FACULTATIVE POND

ANAEROBIC POND

ANAEROBIC POND

Discharge bay

Discharge bay

NEARBY WATER DRAIN

SOLIDS DISCARDED

FOUR DRYING BEDS (not in use)

MATURATION POND

Sludge flow Water flow

Figure S-1. Flowchart of the treatment process implemented at the FSTP in Tema.

Discharge bay (leachate from the nearby landfill)

Discharge bay (leachate from the nearby landfill)

MATURATION POND

ANAEROBIC POND

D (1)

FACULTATIVE POND

ANAEROBIC POND

ANAEROBIC POND

ANAEROBIC POND

Discharge bay

Discharge bay

MATURATION POND

NEARBY WATER DRAIN

ANAEROBIC POND

ANAEROBIC POND

Figure S-2. Flowchart of the treatment process implemented at the FSTP in Kumasi.

MATURATION POND

MATURATION POND

ANAEROBIC POND

ANAEROBIC POND

FACULTATIVE POND

ANAEROBIC POND

ANAEROBIC POND

Discharge bay (fecal sludge)

NEARBY WATER DRAIN

ANAEROBIC POND

ANAEROBIC POND

Discharge bay (fecal sludge)

Discharge bay (leachate from the nearby landfill)

Discharge bay (leachate from the nearby landfill)

Figure S-3. Flowchart of the treatment process implemented at the FSTP in Sekondi-Takoradi.

*Not in use*

Discharge bay (eachate from the nearby landfill)

MATURATION POND

NEARBY WATER DRAIN

FACULTATIVE POND

FACULTATIVE POND

FACULTATIVE POND

FACULTATIVE POND

ANAEROBIC POND

ANAEROBIC POND

Discharge bay (fecal sludge)

Figure S-4. Flowchart of the treatment process implemented at the FSTP in Tamale

Sludge to be processed

Receiving bay

Coarse screening

Fine screening

Regulating tank

Dewatering

Stabilization tank

UASB

Anoxic tank

Aerobic tank

Sedimentation tanks

U.V. Disinfection

Clear water tank

Surface waterbody

Sludge to be processed

Power generation (from Mudor treatment plant)

Figure S-5. Flowchart of the treatment process implemented at the FSTP in Accra

# Key informant questionnaire

**Treatment plants and municipalities’ involvement questionnaire**

**Introduction**

The objective of this questionnaire is to:

- Understand the status of treatment plants in Ghana
- Understand the institutional arrangement around FS treatment
- Collect data to estimate treatment costs in Ghana for fecal sludge (FS)

Once data are collected, an assessment will be conducted to ascertain the support that municipalities may need to improve the treatment performance of FS treatment plants.

This questionnaire is to be addressed to the **FS** plant manager and waste management department heads of target municipalities. These include:

The AMA, TMA, TaMA, SKMA, KMA.

The data are collected by the International Water Management Institute (IWMI).

| Name: | | Address: | |
| --- | --- | --- | --- |
| Telephone: | Email: | | Website: |

***Question 1:*** *How many treatment plants are in existence in your area?*

🞏 1 🞏 2 🞏 3 🞏 Other........................................................

*The treatment plant (s) is (are) principally owned by…………………………………….., a structure that is:*

🞏 Governmental 🞏 Semi-public 🞏 Private 🞏 Other........................................................

🞏 Profit organization 🞏 Non-profit organization

***Question 2:*** *What is the institutional arrangement for the management of the FS treatment plant?*

🞏 100% public O&M 🞏 100% Private O&M 🞏 Other (explain who does what)

***Question 3:*** *If 100% public, do you receive external help to manage the treatment plant?*

🞏 No 🞏 Yes (Please explain what kind of support you receive and when)

***Question 4:*** *Indicate the type(s) of FS treated by the plant.*

🞏 Domestic: ………………% of the total 🞏 Institutional (e.g. public toilets) : ………………% of the total

🞏 Other (specify) ……………………………………… : ………………% of the total

***Question 5:*** *Indicate the type and the measurement (number of ponds or drying beds) of the treatment system in operation.*

🞏 Waste stabilization ponds only 🞏 Waste stabilization ponds + drying beds

🞏 Other

***Question 6:*** *This specific treatment process has been in operation since [year] ………………………*

*What were the main milestones in the O&M of the FS treatment plant (mention rehabilitations, changes in O&M, changes in institutional arrangement, etc.)?*

***Question 7:*** *What is the total amount of FS treated (m^3^ each day)?*

Minimum: ………………………………………………………………………………………………………………………..…… Maximum: ………………………………………………………………………………………………………………………..…..

Average: ………………………………………………………………………………………………………………………..……..

Design: ………………………………………………………………………………………………………………………..………..

***Question 8:*** *What are the design and real performances of the FS treatment plant?*

| Target parameter | Effluent concentration | | Removal rate (%) | |
| --- | --- | --- | --- | --- |
|  | Design value | (Real) average | Design value | (Real) average |
| Total solids |  |  |  |  |
| Organic matter (COD or BOD) |  |  |  |  |
| Ammonia |  |  |  |  |
| Total nitrogen |  |  |  |  |
| Total phosphorus |  |  |  |  |
| Metals ………………………………………. |  |  |  |  |
| Other ………………………………………… |  |  |  |  |

***Question 9:*** *What are the main components of your treatment process?*

Indicate if data below are per 🞏 unit or 🞏 for all.

|  | General description | Number | Electricity consumption | Number of working hours per month/year | Operation cost | Maintenance cost |
| --- | --- | --- | --- | --- | --- | --- |
| Pumps |  |  |  |  |  |  |
| Office equipment(On site) |  |  |  |  |  |  |
| Office equipment(Off site) |  |  |  |  |  |  |
| Generator |  |  |  |  |  |  |
| Other equipment on site (describe) |  |  |  |  |  |  |
| Other equipment off site (describe) |  |  |  |  |  |  |

***Question 10:*** *What use is made of the treated water or FS (give percentages if applicable)?*

🞏 Irrigation] ………% 🞏 Aquaculture ………% 🞏 No use/discharge ………% 🞏 Other .......................

*If applicable, what are the barriers that limit the reuse of treated water? (Examples: Insufficient quantity, insufficient quality, no interested end-user, not feasible) ………………………………………………………………………….*

***Question 11:*** *Do you produce biogas?*

🞏 No 🞏 Yes, but we don’t collect/use it 🞏 Yes, and we use it for …………………………

The current overall amount of biogas is ………………………………. m^3^/d or kg/d

***Question 12:*** *Are you satisfied with your current level of FS treatment?*

🞏 Yes (Please explain) 🞏 No (Please explain how it could be improved and what should be improved)

***Question 13:*** *At what frequency do you follow-up on the FS treatment efficiency (e.g. samples are taken for analysis)?*

🞏 Never 🞏 …………..… times each year/month/week 🞏 Continuously

***Question 14:*** *Do you have staff working at/for the FS treatment plant?*

🞏 No 🞏 Yes (please provide details below)

| Position | Salary band (approx.) per month / year | Role (s) |
| --- | --- | --- |
| Laborer: basic education (explain) | Average ………………  Minimum …………….  Maximum ……………. | Number of full-time workers ………………..  Role (s) of full-time workers  ………………………………………………………………………………………………… |
|  |  | Number of part-time workers ……………….  Workhours per week: Min…….. ; Max ……..; Av. ……………….  Role (s) of part-time workers  …………………………………………………………………………………………………. |
|  |  |  |
| Technician: intermediate education (explain) | Average ………………  Minimum …………….  Maximum ……………. | Number of full-time workers ………………..  Role (s) of full-time workers  …………………………………………………………………………………………………. |
|  |  | Number of part-time workers ……………….  Workhours per week: Min…….. ; Max ……..; Av. ……………….  Role (s) of part-time workers  …………………………………………………………………………………………………. |
|  |  |  |
| Engineer: Advanced education (explain) | Average ………………  Minimum …………….  Maximum ……………. | Number of full-time workers ………………..  Role (s) of full time workers  …………………………………………………………………………………………………. |
|  |  | Number of part-time workers ……………….  Workhours per week: Min…….. ; Max……..; Av. ……………….  Role (s) of part-time workers  …………………………………………………………………………………………………. |
|  |  |  |

***Question 15:*** *What are the key problems that you encounter in the management/operation of the treatment plant?*

🞏 *Technical* *[ex.: electricity shortage, broken pumps] (Provide frequency of occurrence)* ……………..…………

🞏 *Social* …………………………………………………………………………………………………………………………………………………..

🞏 *Economic* …………………………………………………………………………………………………………………………………………….

🞏 *Environmental* …………………………………………………………………………………………………………………………………….

🞏 *Institutional* ……………………………………………………………………………………………………….……………………………….

🞏 *Other* …………………………………………………………………………………………………………….……………………………….……

***Question 16:*** *When a problem occurs, e.g. a technical problem, what is the average response time for a repair to be made? How many people must a maintenance request pass through (explain)?*

- To address a minor problem, …………. days/ months are required. The request passes through …….. people

- To address an average problem, ……. days/ months are required. The request passes through …….. people

- To address a serious problem, ………. days/ months are required. The request passes through ………. people

………………………………………………………………………………………………………………………………………………………………

***Question 17:*** *What are your revenues per year?*

🞏 No revenue

🞏 Tipping fees: …………………………………………………………………………………………………………………………………….

🞏 Others ………………………………………………………………………………………………………………………………………………

***Question 18:*** *What are your other operational costs per year for the FS treatment plant?*

- Electricity cost is ……………………………….………………………………………………………….……………………………………..

- Water cost is …………………………………………….…………………………………………………….……………………………………

- Fuel consumption is ………………………………..…………………………………………………………….……………………………..

- Maintenance (cleaning, protective personal equipment) cost is…………………………………………………………..

- Computers (number and frequency of renewal per year) cost is ………………………………………………………….

- Office cost (printing, travel, calls …) is ………………………………………………………………………………………………….

- Administration cost is ………………………………………………………………………………………………………………………….

- Other costs are (describe) ..…………………………………………………………………………………………….……………………

***Question 19:*** *What does the municipality plan in the future for better maintenance of the FS treatment plant*

# Primary Data collected

Table S-7.Tasks assigned to FSTP staff and their salaries

| **Education levels** | **Tasks** | **Salary band (USD/month)** | | | | |
| --- | --- | --- | --- | --- | --- | --- |
|  |  | **Tema** | **Kumasi** | **Sekondi-Takoradi** | **Tamale** | **Accra** |
| Level 1 (basic school) | - Supervise/direct the trucks when they are desludging - Clean the discharge section - Ensure continuous flow of the wastewater from one pond to the other - Ensure the control structures are periodically rodded - Remove plastic from the screen of the ponds - Weed the premises and its surroundings - Assure security within the FSTP premises - Provide an activity report | Number of employees: 2  Min: 45  Max: 91  Av.: 68 | Number of employees: 3  Min: 45  Max: 91  Av.: 68 | Number of employees: 4  Min: 23  Max: 159  Av.: 91 | Number of employees: 2  Min: 102  Max: 132  Av.: 114 | Number of employees: 57  Min: 152  Max: 248  Av.: 196 |
| Level 5 (Diploma in Water Sanitation or National Vocation Training Institute (NVTI) certificate | - Supervise the laborers - Record and report the number of trucks discharging at the FSTP and write quarterly reports - Supervise the operation of TP for the contractor (if applicable) - Maintain the equipment in good state - Respond to an emergency - Adjust and assure the availability of spare parts (in Accra only) | Number of employees: 2  Min: 121  Max: 204  Av.: 163 | Number of employees: 1  Min: 273  Max: 341  Av.: 307 | Number of employees: 2  Min: 91  Max: 273  Av.: 307 | Number of employees: 3  Min: 148  Max: 166  Av.: 157 | Number of employees: 5  Min: 273  Max: 523  Av.: 364 |
| PhD, MSc or BSc in:   - Civil engineering - Sanitary engineering - Environmental engineering   Water and sanitation | - Supervise and monitor technicians and laborers - Assist and control cost generated at the FSTP, incl. collection of bills of the amount of FS discharged - Ensure that the treated water complies with the disposal standards - Write quarterly and annual reports on the FSTP - Supervise the operation and periodic maintenance of the TP - Communicate with the contractor, if applicable - Manage FSTP-related activities, incl. controlling and organizing labor shifts - Report to the head of the department - Perform/handle any complex activities - Ensure treatment performance targets are met | Number of employees: 3  Min: 207  Max: 645  Av.: 426 | Number of employees: 2  Min: 273  Max: 341  Av.: 307 | Number of employees: 3  Min: 318  Max: 454  Av.: 386 | Number of employees: 1  Min: 216  Max: 364  Av.: 227 | Number of employees: 9  Min: 523  Max: 909  Av.: 682 |

Table S-8. Travel and office costs related to management of FSTPs (in USD)

| **Cost item** | **Details** | **Tema** | **Kumasi** | **Sekondi-Takoradi** | **Tamale** | **Accra** |
| --- | --- | --- | --- | --- | --- | --- |
| Travel | Fuel for motorbikes, cars or bus | 1,254 | 545 | 2,399 | 2,726 | 26,114 |
|  | Motorbike maintenance | 682 | 454 | 1,091 | 382 | 0 |
|  | Car and bus maintenance | 0 | 0 | 2,726 | 477 | 0 |
|  | Other travel (taxi) | 218 | 0 | 0 | 0 | 0 |
| General office | Stationary + phone call allowance | 0 | 0 | 511 | 0 | 149,952 |
|  | Administration (meetings) | 0 | 0 | 256 | 0 | 40,896 |

Table S-9. Detailed operation cost in USD/year of FSTPs in Ghana

| **FSTPs** | **Cost category** | **Item** | **Description** | **Number** | **Energy**  **used** | **Operation cost (USD/year)** |
| --- | --- | --- | --- | --- | --- | --- |
| Tema | Equipment | Personal protective equipment (PPEs) | Mainly used by the on-site workers | Not provided | Not applicable | 4,090 |
|  | General | Water usage | For cleaning purposes | Not applicable | Not applicable | 536 (for water bills) |
| Kumasi | Equipment | Weighing bridge | Weigh the trucks at the entrance of the TP | 1 | In disrepair | - |
|  | General | Water usage | Main water source is a borehole | 1 | Electricity | - |
| Sekondi-Takoradi | Equipment | Electrical pump | Desludge the ponds when full | 1 | Electricity | Part of electricity cost |
|  |  | Weigh bridge | Weigh the trucks before discharging the FS | 1 | Electricity | Part of electricity cost |
|  |  | PPEs | Used by the workers who help with truck  desludging | Not available | Not applicable | 2,726 |
|  | General | Electricity bill | Operation of the weigh bridge and the electrical pump | 1 each | Electricity | 283 |
|  |  | Water usage | For cleaning purposes | - | Not applicable | 1,772 |
|  |  | Effluent sample analysis | Occasionally, samples are collected and analyzed | 1 | Not applicable | 886 (paid by the private sector) |
| Tamale | Equipment | Weighing bridge | Weigh the trucks at the entrance of the TP | 1 | In disrepair | No cost |
|  |  | PPEs | Used by the workers who help with truck  desludging | Not provided | Not applicable | 613 |
|  | General | Water usage | For cleaning purposes | 1 | Not applicable | 409 |
|  |  | Electricity bill | Lighting of the FSTP | - | - | 477 |
| Accra | Equipment | PPEs | Used by the workers who help with truck desludging | 1 | Not applicable | 68,160 |
|  |  | Generator | Standby generator | 2 | Fuel | 10,906 |
|  |  | Forklift | Transport the dewatered sludge from the plant to the drying beds | 1 | Fuel | 5,453 |
|  | General | Water usage | For cleaning purposes | Not available | Not applicable | 95,424 |
|  |  | Electrical appliance | Refrigerator  TV set  Air-conditioner | 15  3  28 | Electricity | 327,168 |

Table S-10. Maintenance cost of FSTPs in Ghana

| **FSTPs** | **Cost category** | **Description** | **Frequency/year** | **Maintenance cost (USD/year)** |
| --- | --- | --- | --- | --- |
| Tema | Equipment | Desludging of the pond: Hire an excavator and repair the valve | 2 | 909 |
|  | General | Clearing the access road to the discharging bay | On demand | Combined with other expenditures such as staff cost |
|  |  | Weeding of the premises: Hire about 20 extra people | 12 | 5,453 |
| Kumasi | General | Spraying of landfill site and adjoining communities (phases 1&2) | 1 | 570 |
|  |  | Weeding of pond berms and surroundings | 1 | 3,408 |
|  |  | Desilting of the drain behind site fence | 1 | 1,036 |
| Sekondi-Takoradi | Equipment | Desludging of the pond: Hire pump | 2 | 218 |
|  | General | Weeding of the premises: Hire a mower | 12 | 436 |
| Tamale | Equipment | Desludging of ponds | 6 | 1,363 |
|  | General | Water tank reservoir cleaning | 1 | 114 |
|  |  | Renewal of the weeding equipment | 1 | 105 |
|  |  | Weeding of pond berms and surroundings | 6 | The cost is accounted with staff cost |
|  |  | Rehabilitation of the access road | 1 | Not available |
|  |  | Desilting of the drain behind the site fence | 1 | Not available |
| Accra | Equipment | Unclogging of the pumps | 58 | 109,056 |
|  |  | Maintenance of the generators | 2 | 9,088 |
|  |  | Maintenance of the forklift | 1 | 5,453 |
|  | General | Maintenance of electrical appliances (TV & refrigerators) | 1 | 2,726 |
|  |  | Maintenance of air-conditioners | 3 | 1,818 |
|  |  | Maintenance cleaning of the plant | 12 | Done by staff. No additional cost |

# Summary of OM&M cost/distribution and revenues of FSTPs in Ghana

Table S-11. Summary of OM&M cost/distribution and revenues of FSTPs in Ghana

| **FSTPs in Ghana** | **Average cost (USD/year)** | **Detailed OM&M cost distribution** | **OM&M cost considering staff salaries, or not average revenue and theoretical profit/loss** |
| --- | --- | --- | --- |
| TEMA | Operation: 4,626 |  | Theoretical profit is:   \| With salaries \| USD 24,293/year \| \| --- \| --- \| \| Without salaries \| USD 45,165/ year \| |
|  | Maintenance: 6,362 |  |  |
|  | Management: 23,026 |  |  |
| KUMASI | Operation: 0 |  | Theoretical profit is:   \| With salaries \| USD 38,589/year \| \| --- \| --- \| \| Without salaries \| USD 52,084/ year \| |
|  | Maintenance:  5,014 |  |  |
|  | Management:  14,495 |  |  |
| SEKONDI TAKORADI | Operation: 5,668 |  | Theoretical loss/profit is:   \| Loss with salaries \| - USD 915/year \| \| --- \| --- \| \| Profit without salaries \| USD 21,714/ year \| |
|  | Maintenance:  654 |  |  |
|  | Management:  29,612 |  |  |
| TAMALE | Operation: 1,500 |  | Theoretical loss is:   \| With salaries \| - USD 17,762/year \| \| --- \| --- \| \| Without salaries \| - USD 6,666/year \| |
|  | Maintenance:  1,581 |  |  |
|  | Management:  14,682 |  |  |
| ACCRA | Operation: 507,110 |  | Theoretical loss is:   \| With salaries \| - USD 846,478/year \| \| --- \| --- \| \| Without salaries \| - USD 617,251/ year \| |
|  | Maintenance:  128,141 |  |  |
|  | Management: 446,190 |  |  |

Abbreviations: a) MAN: Management; b) MAIT: Maintenance; c) OP: Operation; d) Equip: Equipment; e) Gnle: General

# Outcome of the Self-assessment of challenges incurred by the municipalities managing FSTPs

Table S-12. Self-assessment of challenges incurred by the municipalities in managing FSTPs.

| **FSTPs in Ghana** | **Challenges/satisfaction reported** | **Suggestions from municipality** |
| --- | --- | --- |
| Tema | - Occasionally there is no electricity on-site due to power cuts - Malfunction of the valves - Complaints from the neighborhood about the odor from the FSTP - The rate charged (tipping fees) is very low and cannot allow the maintenance of the sustainability of the facility - Possible contamination of the farms and fish ponds downstream with the partially treated effluent - No research institution oversees the physico-chemical analysis of the effluent | TMA plans to:   - Retrofit and upgrade the FSTP - Build a laboratory at the TP in order to evaluate the performance of treatment - Conduct general civil work for maintenance of the site especially at the discharging bay - Upgrade the waste stabilization pond into a more conventional system to produce biogas |
| Kumasi | - The ponds are broken down and are not functioning well - Complaints from the neighborhood about the odor from the FSTP - The rate charged is low and cannot maintain the facility - Quality standards for FSTP effluent are not met | KMA is not satisfied with the current level of FS treatment and expects an immediate renovation of the ponds, or sludge removal because the ponds are completely clogged (i.e. full of sludge). KMA expects to have an additional chamber before the anaerobic pond for screening the FS and to remove the coarse elements. These renovation activities will be funded by one NGO projec |
| Sekondi-Takoradi | - Complaints from the neighborhood about the odor. This phenomenon is pronounced right after the rainy season - Insufficient revenue because the FSTP is underutilized - During the rainy season, overflowing of the ponds was noticed two times in 2017; no major consequences were noticed, and cleaning of drains was performed to ensure a normal flow of the effluent - Delay in the payment of bills for the maintenance of the TP by the contractor | In order to improve the performance of the FSTP, STMA suggests to improve the treatment by introducing activated charcoal treatment. They are also keen to allow the reuse of the treated water for irrigation in agriculture |
| Tamale | - The drains, the weirs, and the leachate chambers are all damaged. - Dysfunction of the weigh bridge. - Complaints from the neighboring community about the potential pollution of the surface water by the effluent. - Inadequate funds for effective operations (no tipping fees taken). - Spills of wastewater during the rainy season (happened in 2017 for the first time). - No autonomy for decision-making when it comes to restoring or improving the management of the TP. | TaMA hopes to:   - Build drying beds to allow recycling of the nutrients in the dewatered FS as fertilizers. - Allocate a laboratory at or for the FSTP in order to monitor the performance of the FSTP |
| Accra | - Breakdown and clogging of pumps - Regular desilting required at the discharging bay - Pressure from the neighborhood for employment - SSGL is satisfied with the current level of treatment because the plant is able to: - Provide a high stock of spare parts - Provide a preventive high maintenance schedule - Spend less time in addressing problems compared to the old New Lavender Hill site - Respond proactively for the management and maintenance of the plant. - Intervene in the process of monitoring | AMA would like to add value to the by-products obtained from the treatment. They believe that treated wastewater or FS could be reused/recycled in agriculture as fertilizer or in aquaculture. The perception is that recycling activities could be financially profitable to AMA and would allow the TP to alleviate the cost of its OM&M |

# References

EPA Ghana, 2000.; *General Environmental Quality standards (Ghana) – Regulations;* **2000**, pp. 8-13

Denutsui, D., Akiti, T.T., Osae, S., Tutu, A.O., Blankson-Arthur, S., Ayivor, J.E., Adu-Kwame, F.N., Egbi, C., 2012. Leachate characterization and assessment of unsaturated zone pollution near municipal solid waste landfill site at Oblogo, Accra-Ghana. *J. Environ Earth Sci.* **2012**, 4(1), 134-141.

Nartey, V.K., Hayford, E.K., Ametsi, S.K., 2012. Analysis of leachates from solid waste dumpsites: a tool for predicting the quality of composts derived from landfills. *J. Environ. Earth Sci.* 2012. 2(11), 8-20.

Nikiema, J., Tanoh, R., Abiola, F., Cofie, O.O., 2020. *Fecal sludge collection, treatment and composting potential in selected West-African cities;* CGIAR Research Program on Water, Land and Ecosystems (WLE), RRR Series, 2020.

Nyame, F.K., Tigme, J., Kutu, J.M., Armah, T.K., 2012. Environmental Implications of the discharge of municipal landfill leachate into the Densu River and surrounding Ramsar wetland in the Accra Metropolis, Ghana. *J. Water Resour. Prot.* 2012, 4, 622-633.

Sackey, L.N.A., Meizah, K, 2015. Assessment of the quality of leachate at Sarbah landfill site at Weija in Accra. *J. Environ. Chem. Ecotoxicol.* 2015, 7(6), 56-61.

Sewerage System Ghana Limited, 2017. Personal communication.

Waste Management Department, Kumasi Metropolitan Assembly, 2017. Personal communication.

Waste Management Department, Sekondi-Takoradi Metropolitan Assembly, 2017. Personal communication.

Waste Management Department, Tamale Metropolitan Assembly, 2017. Personal communication.

Waste Management Department, Tema Metropolitan Assembly, 2017. Personal communication. WHO (World Health Organization), 2011. Guidelines for the safe use of wastewater, excreta and greywater; Vol. 2 Wastewater use in agriculture. WHO. 222p. Available at http: //www.who.int/water_sanitation_health/publications/gsuweg2/en/. Accessed on 15 November, 2018
